# A Preliminary Investigation of Additive Manufacture to Fabricate Human Nail Plate Surrogates for Pharmaceutical Testing

**DOI:** 10.3390/pharmaceutics11060250

**Published:** 2019-05-28

**Authors:** Bruno C. Sil, Avnish Patel, Jonathan M. Crowther, David J. Moore, Jonathan Hadgraft, Stephen T. Hilton, Majella E. Lane

**Affiliations:** 1 School of Human Sciences, London Metropolitan University, 166-220 Holloway Road, London N7 8DB, UK; 2 Department of Pharmaceutics, UCL School of Pharmacy, 29-39 Brunswick Square, London WC1N 1AX, UK; avnish.patel.12@ucl.ac.uk (A.P.); jonathan.hadgraft@btinternet.com (J.H.); s.hilton@ucl.ac.uk (S.T.H.); m.lane@ucl.ac.uk (M.E.L.); 3 JMC Scientific Consulting Ltd., 135 High Street, Egham TW20 9HL, UK; jonathan@jmcscientificconsulting.com; 4 GSK Consumer Healthcare, Skin Health R&D, Weybridge KT13 0DE, UK; david.j.moore@gsk.com

**Keywords:** pharmaceutical analyses, transungual drug delivery, 3D printing, ciclopirox olamine, confocal Raman spectroscopy, drug testing surrogates

## Abstract

In vitro permeation studies using nail clippings or nail plates are commonly used in the development of transungual formulations. However, there are ethical, safety and cost issues associated with sourcing such tissues. Herein, we describe a preliminary approach is described for the design and manufacture of a human nail model surrogate based on 3D printing. To evaluate these 3D printed constructs, nails were mounted in conventional glass Franz cells and a commercial antifungal lacquer formulation containing ciclopirox olamine was applied daily to the surrogate printed surfaces for a period of 14 days. On days 8 and 14, the surfaces of the 3D printed nails were washed with ethanol to remove excess formulation. Confocal Raman spectroscopy (CRS) was used to profile the drug in the 3D printed nail. At the end of the Franz cell studies, no drug was observed in the receptor phase. CRS studies confirmed penetration of the active into the model nails with reproducible depth profiles. Our ongoing work is focused on synthesising commercial and non-commercial printable resins that can replicate the physical and chemical characteristics of the human nail. This will allow further evaluation of actives for ungual therapy and advance the development of the surrogate nail tissue model.

## 1. Introduction

The nail plate can be characterised as a thin (0.25–0.60 mm length), hard, slightly elastic, translucent and convex structure. Formed by approximately 25 layers of dead keratinised flattened cells, the nail plate is tightly bound via numerous intercellular links, membrane-coating granules and desmosomes [1]. An assessment of the efficacy of new formulations for treatment of nail diseases requires the provision of human nails or an appropriate surrogate tissue. However, human nails are difficult to source, variable in quality and their use is associated with ethical and safety issues [2,3,4,5,6].

The emergence of additive manufacture (AM) technologies such as 3D printing has heralded a new era for the design of non-expensive and efficient reproducible polymeric matrices. With printer specifications that can now achieve layer thicknesses of ~25 μm and allied with digital design, 3D desktop printers are becoming powerful tools in pharmaceutical research [7,8,9,10]. In recent years, 3D printing has evolved to develop bioprinting of tissues including skin, bone, cartilage and skeletal muscle [11,12,13]. However, the costs of biocompatible resins allied with the complexities of biological tissues have limited the widespread use of the technology. 

In the present work, the research aimed to develop a suitable polymeric membrane that would simulate the permeation properties of a human nail. When conducting permeation studies with nails, a further problem that researchers face is the natural curvature of the nail. This means that nails are usually flattened, or pressure is applied to the tissue so as to assemble it in a diffusion cell and this may compromise nail integrity leading to microtears and misleading permeation information. A secondary objective of the present study was, therefore, to ensure the 3D printed nails were flat to ensure ease of mounting in diffusion cells. A commercial antifungal lacquer containing ciclopirox olamine (Mycoster 80 mg/g) was selected for in vitro studies with the 3D printed nails.

## 2. Materials and Methods 

### 2.1. Materials

Ciclopirox olamine, anhydrous acetic acid, sodium edetate and acetylacetone were supplied by Sigma Aldrich, Dorset, UK. Phosphate buffer saline (PBS) tablets were purchased from Oxoid Limited, Basingstoke, UK. A commercial formulation of ciclopirox olamine, Mycoster 80 mg/g (Pierre Fabre Dermo-Cosmetique, Paris, France), was purchased from a pharmacy retailer. High performance liquid chromatography (HPLC) grade water and acetonitrile were obtained from Fisher Scientific, Loughborough, UK.

Nail matrices were drawn in silico using the online computer aided design (CAD) program, TinkerCAD (Autodesk, San Rafael, CA, USA). Graphical images of the designs were imported to Preform™ software (version: 2.14.0, Formlabs, Somerville, MA, USA) prior to printing. 3D printing was carried out using two different stereolithography (SLA) machines: Form1+ and Form2 (Formlabs, Somerville, MA, USA). The stereolithography resin used for printing was GPCL04 (Formlabs, Somerville, MA, USA). This is an acrylate-based resin and was selected because it allows preparation of transparent matrices to facilitate visual examination of drug uptake.

### 2.2. 3D Printing Prototype (Design and Printing Parameters)

**Figure 1:**
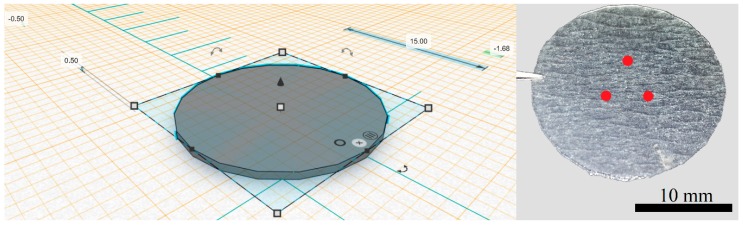
TinkerCad desing of the 3D printed nail (**left**) and image of 3D printed nail printout: red dots signify the approximate locations that were scanned using confocal Raman spectroscopy (**right**).

As shown in Figure 1, the 3D printed polymeric matrix was manufactured with the following specifications and corresponding tolerances: outer diameter (O.D.) = 15.0 ± 1.0 mm and height (*h*) = 0.50 ± 0.10 mm. Printing was carried out using a resolution of 25 μm (conferring ~ 20 unique layers per nail) and a resin tank temperature of 28 °C. In the process of manufacturing 3D printed materials, supports are required for removal of the object from the building platform at the end of the process. Preform™ generated supports were not selected for this study. Instead, the building plate was coated prior to printing with Scotch-Blue™ painter’s tape for multi-surfaces 24 mm (3M, Flemington, NJ, USA) to allow a rapid removal of the printed matrix, preventing the damage of both the printout nail and printing platform. Post-curing of resin was achieved by exposing the 3D printed matrices to UV light (405 nm) at 60 °C for 30 min using the Form Cure (Formlabs, Somerville, MA, USA) [14]. All measurements were done on different areas of the 3D printed scaffolds with an MW110-15WR electronic digital Vernier calipers (Bowers Group, Surrey, UK) and surveyed using Prism7 (Version 7.05, GraphPad Software, San Diego, CA, USA).

### 2.3. High Performance Liquid Chromatographic (HPLC) Analysis

The analysis of ciclopirox olamine was carried out using an HPLC (Agilent Technologies 1200 series) equipped with an Agilent G1322A degasser, G1311A quaternary pump, G1329A auto sampler and G1316A thermostat (Agilent Technologies, Santa Clara, CA, USA). The column used was a Phenomenex Luna CN (nitrile silica gel) column (Phenomenex, Macclesfield, UK). The length, internal diameter and particle size were 120 mm, 4 mm and 5 μm, respectively. To ensure desorption of any interfering metal ions in the stationary phase, the column was rinsed prior to use with a mixture of acetonitrile:water (50:50) containing acetylacetone (0.1%) and anhydrous acetic acid (0.1%) over 15 h and subsequently with mobile phase for 5 h at a flow rate of 0.2 mL/min. The mobile phase consisted of acetonitrile:water (23:77) with anhydrous acetic acid (0.1%) and sodium edetate (0.1%). The mobile phase was degassed using an ultrasonicator (VWR International, Lutterworth, UK) prior to use to remove air bubbles. The flow rate of the mobile phase was 0.7 mL/min and the column temperature set at 23 °C. The chromatogram was acquired at a wavelength of 298 nm. A sample volume of 10 μL was injected for a total run time of 25 min [15] and the drug peak eluted at 12.4 min. A known amount of ciclopirox olamine was dissolved in PBS (pH 7.3 ± 0.1) and a stock solution (2 mg/mL, equivalent to 1.54 mg/mL of ciclopirox) prepared. The stock solution was diluted to prepare a range of concentrations of ciclopirox olamine. The calibration curve was constructed in the concentration range of 0.05-1.54 mg/mL of ciclopirox. A linear relationship was found between concentration and peak area with regression coefficient values (*r*^2^) of greater than 0.99, with a limit of detection (LOD) of 0.025 mg/mL and a limit of quantification (LOQ) of 0.051 mg/mL.

### 2.4. Permeation Study with Commercial Formulation in 3D Printed Polymeric Matrices

The nail printouts were placed in freshly prepared PBS (pH 7.3 ± 0.1) to hydrate for 24 h prior to the start of the experimental work. Permeation studies of the ciclopirox olamine lacquer formulation (Mycoster 80 mg/g) in glass Franz diffusion cells were conducted under finite dose (13 μL/cm^2^) conditions with a daily application to the 3D polymeric matrices for 14 days. The final nails selected for evaluation had a mean application area of 1.13 ± 0.05 cm^2^. The integrity of the polymeric matrices was confirmed by evaluation of electrical resistance as reported previously for topical permeation studies [16]. Freshly prepared PBS (pH 7.3 ± 0.1) was used as the receptor solution since this medium provides sink conditions for the drug. Once the 3D printed nail temperature had equilibrated to 32 ± 1 °C, the formulation was applied using an Eppendorf Multipette Plus. Formulations were applied daily after washing with distilled water to remove residual formulation from the previous day’s application. A volume of 200 μL of receptor solution was removed from the receptor compartment once daily up to 14 days, with sample replacement using fresh temperature equilibrated PBS solution. On days 7 and 14, the 3D printed polymeric surfaces were washed using a cotton tip and distilled water, and a second cotton tip containing a 96% ethanolic solution as reported elsewhere [17]. All samples were analysed using HPLC. The number of replicate experiments was *n* = 4.

### 2.5. Confocal Raman Spectroscopy (CRS) Study of Ciclopirox Olamine in 3D Printed Polymeric Matrices

CRS was performed using a Model 3510 Skin Analyser (RiverD, Rotterdam, Netherlands) with the fingerprint region (400–2200 cm^−1^) using a 785 nm laser. Spectral acquisition and analysis were conducted using software packages, RiverIcon V3.0 and SkinTools V2.0 D2O respectively (RiverD, Rotterdam, Netherlands). Each of the 3D printed nails were cut into smaller fragments and three pieces from each nail were used to profile the drug as shown in Figure 1. A drop of HPLC grade water was placed on the sample window directly above the microscope objective and a piece of 3D printed polymeric matrix (formulation application side facing down) was placed on the window. A stainless-steel weight was placed directly above the 3D printed nail to ensure good contact was obtained between the polymeric printout and microscope window. The piece of 3D printed matrix was scanned using a 20 s exposure time, acquired in 10 μm steps with measurements taken over a total depth of 400 μm. All measurements were taken at room temperature. Spectral data were analysed via Raman band ratios. The 710 cm^−1^ Raman band of ciclopirox was normalised by area to the 1446 cm^−1^ Raman band present in each spectrum using Prism7 (Version 7.05, GraphPad Software, San Diego, CA, USA). 

## 3. Results and Discussion

### 3.1. 3D Printing Prototype (Design and Printing Parameters)

The initial prototyped process for printing 3D nail polymeric matrices was complex since it involved the removal of minor structures from the printer’s building platform without breakage, cracking and/or deformation of the printout. Prior to printing, the inclusion of building supports that could assist in the removal of the printed nails was attempted but dismissed due to poor lithography outcomes. To achieve reproducibility of the printing methodology, the 3D printer’s building platform was then coated using different polymeric materials (Table 1). The Scotch-Blue™ painter’s tape for multi-surfaces allowed for a rapid removal of the printouts from the printing platform which facilitated the development of the desired nail structures.

**Figure 2:**
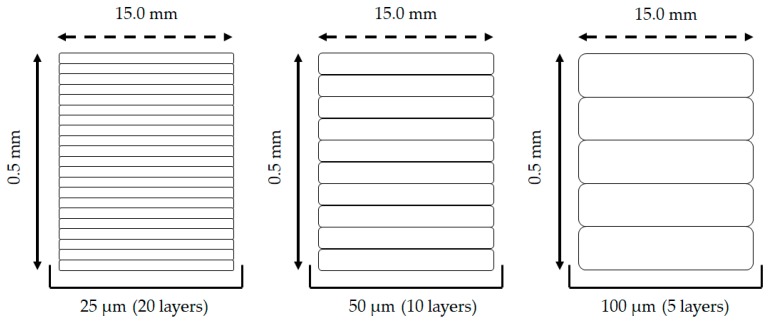
Illustration of the different number of layers present per scaffold using (left to right) 25, 50 and 100 μm printing resolutions.

Printing of new polymeric constructs using the abovementioned platform coating was then obtained in different layer resolutions: 100, 50 and 25 μm, which corresponded to a total sum of ~5, 10 and 20 layers per scaffold respectively (Figure 2). This allowed for a more accurate replication of the human nail layering characteristics (~25 layers). 

Multiple printing attempts which included the manipulation of nail dimensions (i.e., thickness and length), layer resolution and temperature indicated a better printing control for thicker nail structures with less deviation in width throughout the entire nail matrix (Table 2). Throughout these studies, no clear shrinkage of the printout (post-curing) was seen with deviations being attributed to the printing methodology. Optimal printing conditions which rendered the most desirable printout that closely mirrored the nail’s architecture (O.D. = 15 mm; *h* = 0.50 mm; printer resolution: 25 μm; printing temperature = 28 °C) were then used to conduct the permeation study.

### 3.2. Permeation Study with Commercial Formulation in 3D Printed Polymeric Matrices

In vitro testing on nail tissue is limited by the small surface areas of these membranes, approximately 0.03–0.78 cm^2^, when mounted in conventional modified Franz diffusion cells [2,3,18,19,20]. This new approach enabled the printing of polymeric constructs that mimic a typical human nail’s specifications (i.e., number of layers, thickness and diameter) but with the advantage of a wider area for drug application of 1 cm^2^.

After the development of 3D printed nail prototype surrogates, a widely studied antifungal formulation was chosen to test these preliminary polymeric constructs. Permeation studies demonstrated that ciclopirox olamine was not detected in the receptor fluid after 14 days. Previous studies indicate that only small amounts of the active, ranging from 0.25–0.69% of the applied dose, permeated the human nail plate [17,19,20]. Due to the absence of a drug in the receptor phase, CRS was subsequently used to profile drug uptake in the 3D nails.

### 3.3. Confocal Raman Spectroscopy (CRS) Study of Ciclopirox Olamine in 3D Printed Polymeric Matrices

**Figure 3:**
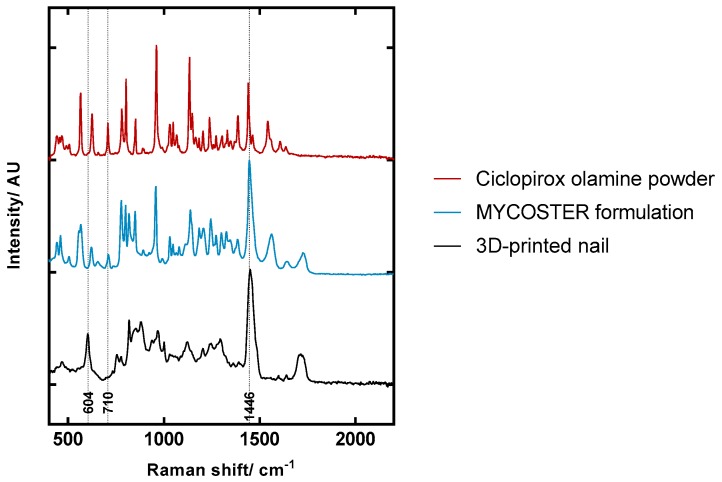
Reference spectra for Ciclopirox olamine, Mycoster^®^ and 3D printed nail.

Representative spectra of ciclopirox olamine, the commercial formulation and the 3D printed polymeric matrix, are shown in Figure 3 with key Raman bands identified. Spectra were scaled and offset for clarity. The 604 cm^−1^ band was only identified in the 3D printed polymeric construct. This peak was used to confirm the position of the surface of the 3D printed nail and was also used as a point to align depth profiles. The 710 cm^−1^ band was present for both ciclopirox olamine and the commercial formulation. Understandably, there are many common peaks between the commercial formulation and ciclopirox olamine, and by comparing the two spectra, it is evident that the 710 cm^−1^ peak is only produced by ciclopirox olamine and not by the formulation excipients. This peak is sharper in the ciclopirox olamine powder spectrum than it is in the commercial formulation because the drug in the formulation is in solution and not that of a pure substance. Raman spectra of crystalline substances are more intense when compared to those obtained in solutions. The 710 cm^−1^ band was selected to detect the penetration of ciclopirox since it did not interfere with any bands produced from the 3D printed nail spectra. Peak areas of this band were normalised to the 1446 cm^−1^ band that is attributed to CH bending [21]. The 1446 cm^−1^ is relatively large in both the 3D printed polymeric matrix and the commercial formulation. Excipients in the formulation, such as isopropyl alcohol, ethyl acetate and the methylvinyl ether-maleic acid monobutylester copolymer all contribute to this spectral feature. This peak is broader in the 3D printed polymeric matrix and there is a distinct lack of sharp peaks is the spectrum, suggesting that the polymer chains matrix have a random arrangement.

**Figure 4:**
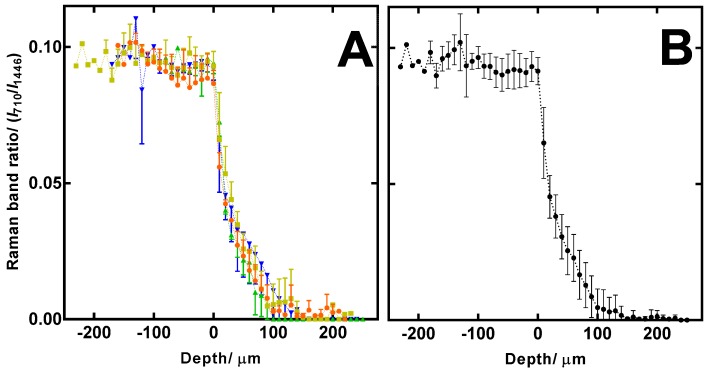
(**A**) Ciclopirox depth profiles in each of the 3D printed nails (*n* = 4) from 3 scans (mean ± SD); (**B**) average ciclopirox depth profile for all 4 nail replicates (mean ± SD).

As shown in Figure 4, ciclopirox content was detected in the residual dried formulation film. CRS measurements also confirmed the mean residual lacquer film thickness as 112.5 ± 45.6 μm. The first frame measured inside the 3D printed nail was taken at 0 μm (Figure 4) and subsequently, deeper measurements were expressed with positive depth values. Depth profiles between replicates were consistent as shown in Figure 4A,B.

Irrespective of the formulation residual film thickness, the concentration of the dried Mycoster 80 mg/g lacquer was the same for each of the 3D printed polymeric matrices (1040 ± 57 μg). A total of 423 ± 36 μg of ciclopirox was found in the residual dried formulation layer after CRS measurements were conducted. The ciclopirox olamine depth profile shown in Figure 4A has a flat portion from -200 μm to 0 μm for each of the experimental replicates. This is because the ciclopirox olamine concentration in the dried lacquer is uniform throughout the depth of this formulation film. The Raman band ratio for ciclopirox olamine in the dried formulation film is constant until scans are acquired from the 3D printed matrix. Scans taken from 0 μm to 25 μm shown a rapid reduction in the ciclopirox olamine concentration. This is because the drug is diffusing from what is effectively a reservoir of drug in the dried film into the 3D printed matrix (i.e., a high drug concentration movement into the polymeric matrix that has a drug concentration of zero). From approximately 25 μm to 100 μm, the depth profile becomes shallower and shows ciclopirox olamine is accumulating in the deeper portion of the 3D printed matrix. Only one of the replicates showed a detectable amount of ciclopirox olamine below 200 μm from the surface of the polymeric matrix. The relatively flat portion of the depth profile from 150 μm to 200 μm confirms that there is no ciclopirox olamine at this depth and explains why ciclopirox olamine was not detected in the receptor fluid during the permeation experiments. It is clear from Figure 4A that the 3D printed nail is a highly reproducible material as each replicate matrix produces a depth profile that is comparable in shape.

## 4. Conclusions

The use of polymeric matrices that not only mimic chemical, but also physical and anatomical properties of the human nail would address the problems researchers face when sourcing such tissues for research and drug development. This study confirms reproducible nail penetration profiles for the model actively studied. Necessary fine-tuning of printing parameters allied with the synthesis of new polymeric resins will allow for more control of the composition and porosity of the nail scaffold going forward.

Current and future work is focused on modifying commercial resins and synthesising novel polymers that incorporate the specific chemistry of the human nail namely, disulfide bonds. Furthermore, it is hoped that modification and diversification of our current 3D printing methodology can achieve different levels of polymer porosity that can translate into diseased nail models. However, the existing experimental model in this study requires vast lengths of time (4–5 weeks) to allow for both 3D printed constructs and single molecular permeation profiles to be characterised and evaluated. Still, we are currently examining the permeation properties of other pharmaceutical ingredients used in ungual preparations with accompanied permeation study methodology updating to accommodate these 3D models.

## Figures and Tables

**Table 1:** Building platform coatings used during the 3D printing process.

Platform Coating	Removal of Printout from Building Platform	Inspection of 3D Printed Scaffold ^1^ (Mean ± SD)
None ^2^	Printout breakage and platform damage on attempt	*h* (0.51 ± 0.02 mm) O.D. (-) ^3^
White masking tape 50 mm (Eurocel Ltd., Alfreton, UK)	Accessible removal of the printout from the plate. Extended peeling of the coating during printing	*h* (0.28 ± 0.22 mm) O.D. (9.3 ± 5.5 mm)
Blue masking tape HB 850 12 mm (Hi-Bond Tapes Ltd., Corby, UK)	Printout did not adhere to the coating	-
Kapton tape 50 mm (3D FilaPrint, Essex, UK)	Unmanageable removal of the coating from the plate. Hindrance to detach printout from coating	*h* (0.48 ± 0.03 mm) O.D. (15.0 ± 0.01 mm)
Scotch-Blue™ painter’s tape for multi-surfaces 24 mm (3M, Flemington, NJ, USA)	Accessible removal of the printout from the plate. Simple separation of the printout from coating	*h* (0.50 ± 0.01 mm) O.D. (15.0 ± 0.02 mm)

^1^ Printing replicates are shown as *n* = 27 (Nine nails per each of three printing batches). ^2^ Printing done directly on the building plate. ^3^ Measurement was not attained due to breakage of the printout.

**Table 2:** Printing conditions (temperature and resolution) for the development of different 3D printed nails scaffolds (*n* = 18).

3D Nail Thickness (mm)	3D Nail Length (mm)	Printing Temperature (°C)	Printing Resolution (μm)	Inspection of 3D Printed Scaffold (Mean ± SD)
0.50	15.0	28	100	*h* (0.50 ± 0.01 mm); O.D. (15.0 ± 0.1 mm)
			50	*h* (0.50 ± 0.01 mm); O.D. (15.1 ± 0.2 mm)
			25	*h* (0.50 ± 0.01 mm); O.D. (15.0 ± 0.2 mm) ^1^
		20	100	*h* (0.50 ± 0.01 mm); O.D. (15.1 ± 0.1 mm)
			50	*h* (0.51 ± 0.01 mm); O.D. (15.0 ± 0.1 mm)
			25	*h* (0.51 ± 0.02 mm); O.D. (15.3 ± 0.2 mm)
	20.0	28	100	*h* (0.50 ± 0.01 mm); O.D. (20.1 ± 0.1 mm)
			50	*h* (0.51 ± 0.01 mm); O.D. (20.3 ± 0.2 mm)
			25	*h* (0.51 ± 0.02 mm); O.D. (20.3 ± 0.2 mm)
		20	100	*h* (0.51 ± 0.01 mm); O.D. (20.2 ± 0.1 mm)
			50	*h* (0.51 ± 0.02 mm); O.D. (20.3 ± 0.2 mm)
			25	*h* (0.52 ± 0.02 mm); O.D. (20.3 ± 0.2 mm)
0.30	15.0	28	100	*h* (0.32 ± 0.01 mm); O.D. (15.0 ± 0.1 mm)
			50	*h* (0.32 ± 0.02 mm); O.D. (15.0 ± 0.1 mm)
			25	*h* (0.29 ± 0.01 mm); O.D. (15.1 ± 0.2 mm)
		20	100	*h* (0.32 ± 0.02 mm); O.D. (15.1 ± 0.1 mm)
			50	*h* (0.29 ± 0.02 mm); O.D. (15.1 ± 0.2 mm)
			25	*h* (0.29 ± 0.02 mm); O.D. (15.1 ± 0.2 mm)
	20.0	28	100	*h* (0.31 ± 0.02 mm); O.D. (20.1 ± 0.1 mm)
			50	*h* (0.32 ± 0.01 mm); O.D. (20.3 ± 0.2 mm)
			25	*h* (0.32 ± 0.02 mm); O.D. (20.3 ± 0.2 mm)
		20	100	*h* (0.33 ± 0.02 mm); O.D. (20.1 ± 0.2 mm)
			50	*h* (0.28 ± 0.03 mm); O.D. (20.2 ± 0.3 mm)
			25	*h* (0.28 ± 0.02 mm); O.D. (20.3 ± 0.2 mm)

^1^ Conditions applied for the manufacture of the 3D printed “nail” scaffold used in the pharmaceutical formulation permeation study.
